# A new species of *Isospora* (Apicomplexa: Eimeriidae) from Carolina chickadee (Passeriformes: Paridae), from Oklahoma, USA

**DOI:** 10.1007/s11230-021-09999-9

**Published:** 2021-09-05

**Authors:** Chris T. McAllister, R. Scott Seville

**Affiliations:** Science and Mathematics Division, Eastern Oklahoma State College, Idabel, OK 74745, USA; Department of Zoology and Physiology, University of Wyoming, Casper, WY 82601, USA

## Abstract

The Carolina chickadee, *Poecile carolinensis* Audubon is a relatively small songbird belonging to the tit and chickadee family Paridae. Feces from three *P. carolinensis* from Polk County, Arkansas, USA, and a single *P. carolinensis* in McCurtain County, Oklahoma, USA, were collected and examined for coccidia; the latter bird was found to be passing a new species of *Isospora*. Oöcysts of *Isospora oklahomaensis* n. sp. are subspheroidal to ovoidal with a smooth to slightly-pitted bi-layered wall, measure (L × W) 32.1 × 28.3 μm, and have a length/width (L/W) ratio of 1.1; a micropyle and oöcyst residuum were absent but a bilobed and refractile polar granule is present. Sporocysts are ellipsoidal and measure 18.4 × 11.8 μm, L/W 1.6; a prominent Stieda body is present as well as a distinct sub-Stieda body. The sporocyst residuum is composed of an irregular mass of granules lying between and dispersed among the sporozoites. This is the first coccidian described from the Carolina chickadee and, most importantly, only the second described from a member of the Paridae, worldwide.

## Introduction

The Carolina chickadee or Mésange de Caroline, *Poecile carolinensis* (Audubon) is a relatively small passerine belonging to the tit and chickadee family Paridae. It possesses a black cap and bib with white cheeks and formerly belonged to the genus *Parus* (L.) but was transferred to *Poecile* Kaup by AOU (1998). This bird ranges (as a permanent resident) from southern Kansas east to central Indiana, southern Pennsylvania, and central New Jersey south to southern Texas, the Gulf Coast, and the northern peninsular of Florida. Four subspecies are recognized (Avibase, 2021), and in Oklahoma, *P. carolinensis atricapilloides* (Lunk) is found statewide except for the Panhandle. It seldom decends to the ground and prefers valleys and foothills in open deciduous woodland, forest clearings and edge, swamps, thickets, second-growth woodland, parks, brushy areas,and suburban areas. Carolina chickadees eat insects, especially moths and caterpillars, insect eggs, spiders, fruits, and seeds (Terres, 1980).

Although there are many species of coccidian parasites reported from other related passerines (see Duszynski et al., 2000; Berto et al. 2011), there are no reports of coccidia from this bird. Here, we describe a new species of *Isospora* from a *P. carolinensis* from Oklahoma, USA.

## Materials and methods

Fresh feces were collected from 3 *P. carolinensis* taken with a mist net in March and May 2021 from the Ouachita Mountains Biological Station (OMBS), Polk County, Arkansas, USA (34° 27′ 43.4484″N, −93° 59′ 54.3264″W). In addition, fresh feces were collected from a single (nesting female) *P. carolinensis* taken from a bird box in April 2021 in Hochatown, McCurtain County, Oklahoma, USA (34°10′17.0286″N, −94°45′05.7414″W); after defecation, all birds were released unharmed. Fecal samples were placed in individual vials containing 2.5% (w/v) aqueous potassium dichromate (K_2_Cr_2_O_7_). After flotation in Sheather’s sugar solution (specific gravity = 1.30), they were examined for coccidia using an Olympus BX53 light microscope (Olympus Corporation, Center Valley, Pennsylvania, USA). One bird was found to be passing unsporulated and partially sporulated oöcysts and the sample was placed in a Petri dish containing a small layer of K_2_Cr_2_O_7_ for 48‒72 h to allow sporulation. It was further examined using Olympus© cellSens 1.14 digital software (https://www.olympus-lifescience.com/en/software/cellsens/) and all morphological measurements are reported in micrometers (μm) with the means followed by the ranges in parentheses. Photographs were taken using Nomarski interference-contrast optics at ×1,000 magnification. Oöcysts were ca. 30 days old when measured and photographed.

Descriptions of oöcysts and sporocysts follow the standard guidelines of Wilber et al. (1998). The host photovoucher was accessioned into the Eastern Oklahoma State College (EOSC) Collection, Idabel, Oklahoma, USA. Photosyntypes of sporulated oöcysts were accessioned into the Harold W. Manter Laboratory of Parasitology (HWML), Lincoln, Nebraska, USA.

## Results

A single Carolina chickadee was found to be passing a coccidian that we describe herein as new.

*Eimeriidae* Minchin, 1903

*Isospora* Schneider, 1881

Isospora oklahomaensis n. sp.

### Type species:

Isospora rara Schneider, 1881 by monotypy.

### Type-and only host:

*Poecile carolinensis* (Audubon, 1834) (Aves: Passeriformes: Paridae), adult female collected 15 April 2021.

### Type-and only locality:

Hochatown, McCurtain County, Oklahoma, USA (34°10′17.0286″N, −94°45′05.7414″W).

### Type-material:

Photosyntypes of sporulated oöcysts are deposited as HWML 216542.

### Prevalence:

1 of 4 (25%) overall; 1/1 McCurtain County, Oklahoma, USA; 0/3 (0%) Polk County, Arkansas, USA.

### Sporulation time:

All oöcysts were fully sporulated within 48‒72 hrs.

### Site of infection:

Unknown; oöcysts were passed in feces.

### ZooBank registration:

To comply with the regulations set out in article 8.5 of the amended 2012 version of the International Code of Zoological Nomenclature (ICZN, 2012), details of the new species have been submitted to ZooBank. The Life Science Identifier (LSID) for *Isospora oklahomaensis* n. sp. is urn:lsid:zoobank.org:act:BD2187AA-9ED5–4DE3–8ABF-F0B4470814BB.

### Etymology:

The specific name is derived from the US state locality for the coccidian, Oklahoma, the 46th state to enter the union on November 16, 1907. The state’s name is derived from the Choctaw words *okla* and *humma*, meaning “honored people”. The new name is formed as an adjective, feminine.

**Fig. 1:**
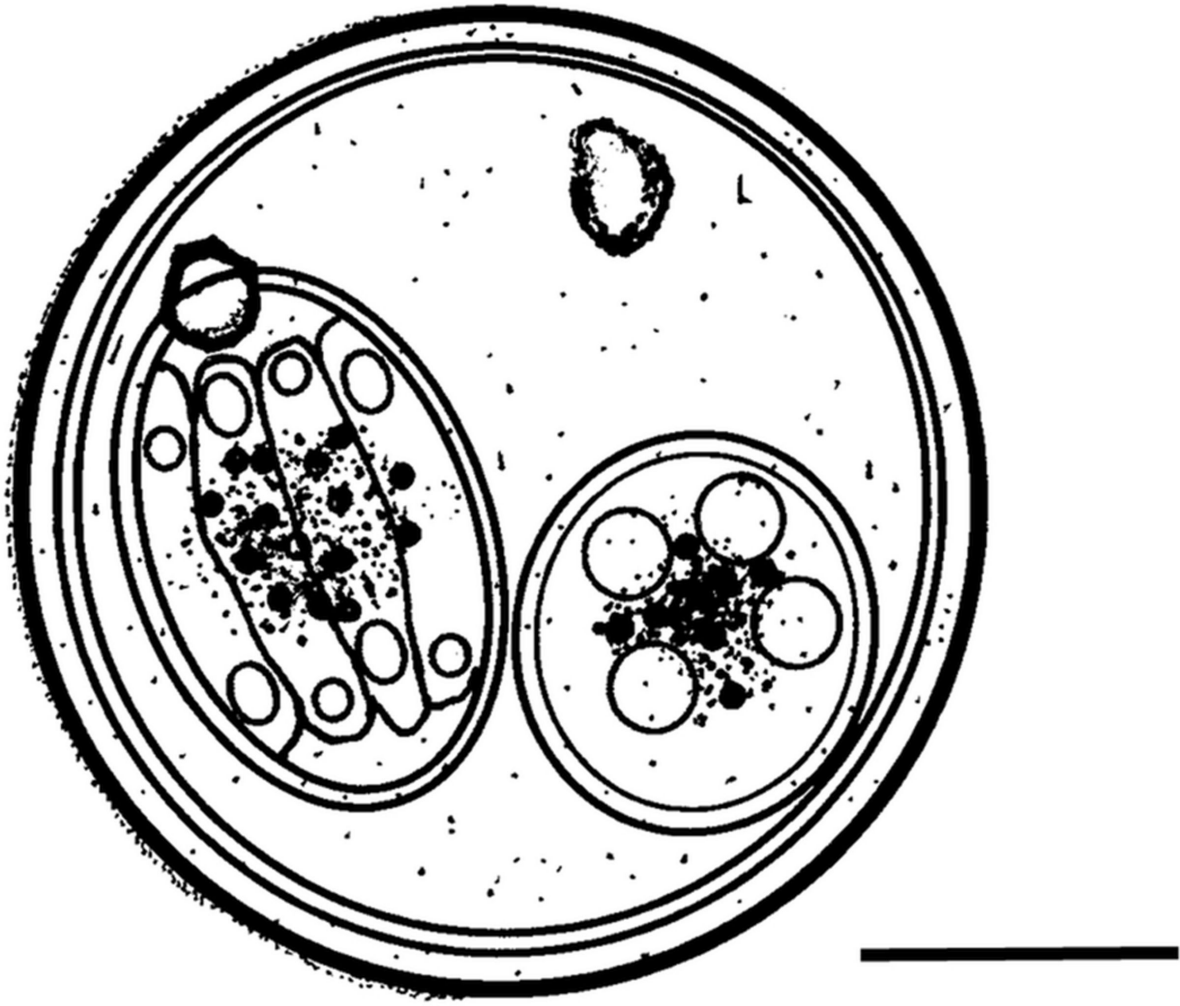
Composite line drawing of ***Isospora oklahomaensis* n. sp.** *Scale bar*: 10 μm

**Fig. 2:**
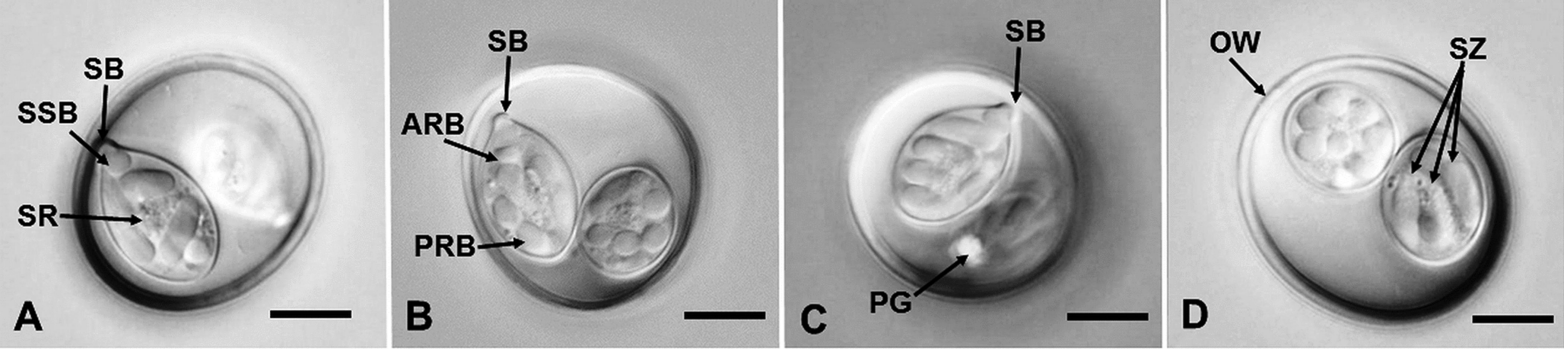
**A-D** Nomarski-interference contrast photomicrographs of sporulated oocysts of ***Isospora oklahomaensis* n. sp. (A)** View showing sporocyst residuum (SR), Stieda body (SB), and subStieda body (SSB). **(B)** Another view showing anterior refractile body (ARB), posterior refractile body (PRB), and SB. **(C)** Oocyst showing polar granule (PG) and SB. **(D)** Oocyst showing oocyst wall (OW) and sporozoites (SZ). *Scale bars*: 10 μm.

Description (Figs. 1, 2A–D)

## Sporulated oöcyst

Oöcyst (n = 15) subspheroidal to ovoidal; (32.1 × 28.3) 29–36 × 27–30, length/width (L/W) ratio 1.1–1.2. Wall smooth to slightly-pitted, thick, bilayered, tan to yellow, c.2.0 (1.3–2.1), outer c. 1.2 (0.8‒1.4), inner 0.7 (0.5‒0.8). Micropyle and oöcyst residuum absent but single bilobed and refractile polar granule (3 × 2.7) present.

## Sporocyst and sporozoite

Sporocysts (n = 15) 2, ovoidal 18.4 × 11.8 (16–20 × 9–13); L/W ratio 1.6 (1.4–1.7); wall smooth, thin and uni-layered, light brown, c.0.5 thick. Prominent Stieda and distinct sub-Stieda bodies present, para-Stieda body absent; sporocyst residuum spheroidal granules of various sizes dispersed between sporozoites. Sporozoites (4), vermiform 12.7 × 3.6; L/W ratio 3.3; subspheroidal anterior and ellipsoidal posterior refractile bodies, nucleus in centre of sporozoites.

## Remarks

There are seven species of the genus *Poecile* Kaup currently recognized in the tit family Paridae Vigors (Gosler and Clement, 2007; AOS, 2020). Surprisingly, only a single coccidian, *Isospora parusae* Ray, Shivnani, Ommen, and Bhaskaran, 1952 from grey-crested tit, *Lophophanes dichrous* (Blyth) from India has been described worldwide from birds of this family (Ray et al., 1952) (Table 1). Furthermore, to our knowledge, there are no coccidian taxa fully described from any member of the Paridae in North America. However, there are several isosporans from this family but none have been described beyond being reported as an *Isospora* sp. (see Table 2).

Nearly all of the older descriptions of bird coccidians (isosporans) from passeriform birds are problematic as they were classified as *Isospora lacazei* (Labbé, 1893), some other combination, or simply as *Isospora* sp. (Table 2). In addition, others have reported oöcyst measurements and photomicrographs of unnamed isosporans from two species of parid birds, Eurasian blue tit, *Cyanistes caeruleus* (L.) and great tit, *Parus major* (L.) from the Czech Republic (Svobodová, 1994; designated types 25 and 26, see her figs. 25‒26, Table 2) and oöcyst and sporocyst measurements of *C. caeruleus*, *P. major*, and willow tit, *Parus montanus* (von Baldenstein) from Great Britain (Brown et al., 2010, Table 2). However, these authors have simply designated these “forms” as *Isospora* sp. in these two documents, so there were no type specimens, line drawings, photosyntypes, stages in tissue sections, and/or oöcysts in preservative, so these isosporans must be considered *species inquirendae*. Furthermore, Svobodová (1994) and Brown et al. (2010) thought that these isosporans were “probably or indicated” new species but, unfortunately, they were never described.

When the new species is compared to *I. parusae*, there are three major differences: (1) oöcysts of the new species are considerably larger (32.1 × 28.3 *vs.* 24.2 × 20.8), (2) *I. parusae* possesses a micropyle that the new species clearly does not, and (3) the hosts are from different, widely-separated continents. In addition, when compared to coccidians reported from New World passerine birds of the Superfamily Sylvioidea (Hirundinidae, Paridae, Timaliidae, Zosteropidae) (Berto et al. 2011), oöcysts of the new species are the largest known from this taxon (Table 1). Therefore, we believe it is clear that ***I. oklahomaensis*** is a new species.

## Discussion

Svobodová (1994) surveyed 13 additional parid birds, including six marsh tits, *Parus palustris* (L.), six *P. montanus*, and a single coal tit, *Parus ater* (L.) from the Czech Republic but none were passing coccidians at the time they were examined. It is obvious that novel coccidian parasites have been rarely described as novel species among members of the Paridae. This is an enigma because this family is a large and widespread group of 64 species of small passerine birds which mainly occur in Europe, Asia, North America, and Africa (AOS, 2020). Therefore, in the past, there should have been plenty of opportunity for these birds to be surveyed for coccidia and be reported as hosts compared to other passeriform species with fewer species serving as hosts of coccidians. Herein, we document the first coccidian from *P. carolinensis* as well as the first described from a parid bird in the Western Hemisphere.

## Figures and Tables

**Table 1:** Comparison of the sporulated oocysts of *Isospora* spp. from Old and New World passerine birds of the Superfamily Sylvioidea (Hirundinidae, Paridae, Timaliidae, Zosteropidae).

*Isospora* spp.	Type host (Describer) Type locality	Oocyst shape, size, features^a,b^	Sporocyst shape, size, features^a,b^	References

*I. brayi*	*Zosterops japonicus* Temminck & Schlegel Hawaii, USA	Subspheroidal 27 × 26; L/W 1.0 26–28 × 25–27 M, OR, PG: all ‒	Ovoidal to pyriform 19 × 12; L/W 1.6 18–21 × 11–13 SB, SSB, SR: all + PSB: –	Upton et al. (1988)
*I. leiothrixi*	*Leiothrix lutea* (Scopoli) Hawaii, USA^c^	Ellipsoidal 28.0 × 16.6; L/W 1.5 24–32 × 15–18 M, OR: both – PG: +	Ovoidal 15.5 × 10.3; L/W 1.5 12–20 × 7–12 SB, SSB SR: all + PSB: –	McQuistion et al. (1996)
*I. manoaensis*	*Z. japonicus* Hawaii, USA	Subspheroidal 28.0 × 26.5; L/W 1.1 25–31 × 22–29 M, OR: both ‒ PG: +	Ovoidal 18.5 × 12.0; L/W 1.5 16–20 × 10–14 SB, SSB, SR: all + PSB: –	Upton et al. (1988)
*Isospora mejiro*	*Z. japonicus* Hawaii, USA	Subspheroidal 28.5 × 27.0; L/W 1.1 25–32 × 25–30 M, OR: both ‒ PG: +	Ovoidal 17 × 11; L/W 1.5 16–19 × 10–12 SB, SSB, SR: all + PSB: –	Upton et al. (1988)
*I. oklahomaensis* n. sp.	*Poecile carolinensis* (Audubon) Oklahoma, USA	Subspheroidal to ovoidal 32.1 × 28.3; L/W 1.1 29–36 × 27–30 M, OR: both ‒ PG: +	Ovoidal 18.4 × 11.8; L/W 1.6 16–20 × 9–13 SB, SSB, SR: all + PSB: ‒	This report
*I. parusae*	*Lophophanes dichrous* (Blyth) India	Subspheroidal 24.2 × 20.8; L/W 1.2 23–28 × 20–23 M, PG: both + OR: ‒	Pyriform 15 × 10; L/W 1.5 10–18 × 10 SB, SR: both + SSB, PSB: both –	Ray et al. (1952)
*I. petrochelidon*	*Petrochelidon pyrrhonota* (Vieillot) Colorado, USA	Subspheroidal to ovoidal 25.2 × 22.2; L/W 1.1 23–30 × 19–25 M, OR: both ‒ PG: +	Lemon-shaped 18.4 × 10.8; L/W 1.7 16–22 × 10–12 SB, SSB, SR: all + PSB: –	Stabler & Kitzmiller (1972)

a Measurements in μm.

b Descriptions of oöcysts and sporocysts follow guidelines of Wilber et al. (1998) as follows: oocyst length (L) and width (W), their ranges and ratios (L/W), micropyle (M), oöcyst residuum (OR), polar granule(s) (PG), sporocyst (SP) length (L) and width (W), their ratio (L/W), Stieda body (SB), substieda body (SSB), parastieda body (PSB), and sporocyst residuum (SR).

c Fecal samples were collected from native birds that originated from Hawaii and captives housed at the Dallas Zoo, Texas, USA.

**Table 2:** Isosporans (as *Isospora* sp.)^a^ reported from members of the family Paridae.

Host (Describer) & Common Name	Locality	References

*Baeolophus inornatus* (Gambel) (Oak Titmouse)	California, USA	Boughton et al. (1938)
*Cyanistes cyanus* (Pallus) (Azure Tit)	France	Labbé (1893, 1896, 1899)^b^
*Cyanistes coeruleus* (L.) (Eurasian Blue Tit)	France; Czech Republic	Labbé (1893, 1896)^b^; Ryšavý (1954)^c^; Boughton et al. (1938); Svobodová (1994)^d^
	Great Britain	Brown et al. (2010) ^e^
*Parus major* (L.) (Great Tit)	Germany; Czech Republic	Wasielewski (1904) ^f^
	Czech Republic	Ryšavý (1954)^c^; Schwalbach (1959)^g^
	Czech Republic	Svobodová (1994) ^d,h^
	Great Britain	Brown et al. 2010)^e^
*Peripatus ater* (L.) (Coal Tit)	Czech Republic	Ryšavý (1954)^c^
*Poecile atricapilla* (L.) (Black-capped chickadee)	Czech Republic	Ryšavý (1954)^c^
*Poecile montanus* (von Baldenstein) (Willow Tit)	Great Britain	Brown et al. (2010)^e^

a All of these isosporans are considered *species inquirendae*.

b Synonym: *Diplospora rivoltae* Labbe, 1893, *pro parte*.

c non *Diplospora lacazei* of Ryšavý, 1954.

d *Isospora* sp. 25 of Svobodová, 1994.

e *Isospora* sp. (Brown et al., 2010).

f Synonym: *Diplospora lacazei* of Wasielewski, 1904.

g non *Diplospora sylvianthina* Schwalback, 1959.

h *Isospora* sp. 26 of Svobodová, 1994.
